# Lifestyle Habits and Anthropometric Indicators Associated with Mediterranean Diet Adherence in Spanish Youth

**DOI:** 10.3390/nu17101718

**Published:** 2025-05-19

**Authors:** Enric Conesa-Milian, Saül Aixa-Requena, Alvaro Pano-Rodriguez, Vicenç Hernández-González, Abraham Batalla-Gavaldà, Jose Vicente Beltran-Garrido, Carme Jové-Deltell, Joao Jose Albuquerque de Sousa Junior, Joaquin Reverter-Masia

**Affiliations:** 1 Department of Specific Didactics, Faculty of Education, Psychology and Social Work, University of Lleida, 25003 Lleida, Spain; saul.aixa@udl.cat (S.A.-R.); alvaro.depano@udl.cat (A.P.-R.); vicenc.hernandez@udl.cat (V.H.-G.); carme.jove@udl.cat (C.J.-D.); joaquim.reverter@udl.cat (J.R.-M.); 2 Human Movement Research Group (RGHM), University of Lleida, Plaça de Víctor Siurana, 25003 Lleida, Spain; 3 University School of Health and Sport (EUSES), Universitat Rovira i Virgili, 43870 Amposta, Spain; a.batalla@euseste.es; 4 Department of Education and Specific Didactics, Faculty of Humanities and Social Sciences, Universitat Jaume I, 12071 Castellón de la Plana, Spain; 5 Physical Exercise and Performance Research Group, Universidad Cardenal Herrera-CEU, CEU Universities, 12006 Castellon de la Plana, Spain; jose.beltrangarrido@uchceu.es; 6 Department of Education Science, School of Humanities and Communication Sciences, Universidad Cardenal Herrera-CEU, CEU Universities, 12006 Castellon de la Plana, Spain; 7 Centro de Ciências da Saúde e do Desporto, Universidade Federal do Acre, Rio Branco 69920-900, Brazil; joao.jose@ufac.br

**Keywords:** Mediterranean diet, pubertal status, adolescents, children, lifestyle factors

## Abstract

**Background**: The increasing prevalence of overweight and obesity among young people poses a significant public health challenge. Childhood and adolescence are critical periods for establishing long-term health habits. Diet plays a central role in maintaining a healthy weight, and adherence to the Mediterranean diet has been consistently linked to numerous health benefits, including improved weight management and overall well-being. Understanding the factors that influence Mediterranean diet adherence in adolescents can help design effective interventions to promote healthier eating behaviors. **Objectives**: This study sought to explore the association between following the Mediterranean diet and key physiological factors, including age, sex, body mass index, and pubertal status. Additionally, it explored how Mediterranean diet adherence relates to lifestyle behaviors such as sleep quality and physical activity levels, using data from a sample of 668 Spanish adolescents. **Method**: Participants completed validated questionnaires assessing dietary habits, physical activity, sleep quality, and pubertal status (Tanner scale). **Results**: The findings revealed significant associations between Mediterranean diet adherence and body mass index, pubertal status, and physical activity level, while sex, age, and sleep quality showed no significant influence. Adolescents with lower body mass index tended to adhere more to the Mediterranean diet, reinforcing its role in weight management. Additionally, greater adherence was observed in later pubertal stages, suggesting increased nutritional awareness. **Conclusions**: Healthy eating patterns are associated with a strong promotion of physical activity, highlighting the link between an active lifestyle and adherence to the Mediterranean diet. This positive multifactorial synergy requires further research to better understand its mechanisms and to design effective strategies for promoting healthy habits among adolescents.

## 1. Introduction

Childhood and adolescence are critical periods for establishing long-term health habits. In recent years, the global prevalence of overweight and obesity in these populations has risen to critically concerning levels [1,2]. According to data from the World Health Organization (WHO), approximately 37 million children under the age of five were classified as overweight. Among children and adolescents aged 5 to 19, over 390 million were overweight, with 160 million living with obesity. About this disease, a high body mass index (BMI) is a significant risk factor that increases the likelihood of suffering from cardiovascular disorders [3]. These data highlight the growing impact of excess weight on younger populations and the urgent need for preventive strategies, as children and adolescents with obesity were approximately five times more likely to remain obese in adulthood compared to their non-obese peers [4].

The Mediterranean diet (MD), characterized by a high consumption of whole grains, legumes, fresh fruits, vegetables, and olive oil, along with a moderate intake of dairy and fish, has gained recognition as one of the healthiest dietary patterns. Research has demonstrated the effectiveness of the MD in reducing obesity-related risk factors and improving the overall quality of life [5]. It has been shown to have beneficial effects on cardiovascular and metabolic diseases, as well as cancer, with its anti-inflammatory and antioxidant properties playing a key role [6,7]. Adolescents who adhere to the MD exhibit better physical and mental well-being, along with improved longevity [8]. The KIDMED questionnaire (Mediterranean Diet Quality Index for children and teenagers) is widely accepted as the most validated tool for assessing adherence to the MD in children and adolescents [9,10].

Engaging in regular PA during childhood and adolescence is essential for fostering overall health and well-being [11]. PA helps in the development of strong bones and muscles, enhances cardiovascular fitness, and promotes healthy weight management [12]. Additionally, active children are more likely to maintain a healthy lifestyle into adulthood, leading to a reduced risk of chronic conditions such as obesity, cardiovascular disease, and diabetes [4].

The declining adherence to the Mediterranean lifestyle suggests a growing shift away from this dietary pattern, which could unfortunately lead to negative health consequences [13]. Special attention should be given to children and young people, as they are showing a clear tendency to abandon this diet at an accelerated rate [14]. Moreover, current levels of PA and physical fitness (PF) among children and adolescents are alarmingly declining, starting as early as preschool and continuing through adolescence. Boys consistently show higher PA and PF levels than girls across all age groups [15]. Given that PA and PF are key factors in improving health-related quality of life among the general population of children and adolescents [12]. Implementing public health strategies to promote healthy behaviors is crucial for enhancing overall well-being and preventing chronic diseases.

Compliance with the MD among children and adolescents is influenced by various factors, including family eating habits, parental education level, availability of healthy foods, socioeconomic status, cultural background, personal preferences, nutritional knowledge, and exposure to advertising and social media [16,17]. Additionally, sleep patterns and screen time seem to play significant roles; for instance, there is a review that discusses the relationship between adherence to the MD and sleep quality, showing favorable effects [18]. Furthermore, the school environment, including the quality of school meals and nutrition education programs, significantly impacts dietary habits. To enhance adherence to the MD in this demographic, it is essential to involve families, promote nutritional education, ensure access to healthy foods, encourage adequate sleep, and manage screen time effectively [13].

By embracing an interdisciplinary and multifactorial lens, this study enriches health and nutrition research through its examination of MD adherence among children and adolescents. Beyond individual factors, it explores the intricate interplay between variables like BMI, pubertal development, PA, and various demographic and lifestyle elements to uncover synergistic relationships with dietary habits. This holistic strategy delivers a more expansive insight into how diet is intertwined with physiological growth and well-being behaviors. Despite numerous investigations focusing on MD adherence or PA in isolation, a comprehensive evaluation of their collective impact alongside anthropometric measures and pubertal stage remains limited, underscoring the distinctive value of this investigation in the context of current literature. The purpose of the present study is to assess adherence to the Mediterranean diet based on sex, age, BMI, pubertal status, sleep quality, and physical activity levels.

## 2. Materials and Methods

### 2.1. Subjects and Design

The study is a descriptive, quantitative, and cross-sectional research carried out in 2023 using a convenience sample selected based on the accessibility and availability of participants, as it assessed adherence to the MD along with different variables at a specific point in time. For the study, participants were invited to a location in their community, where the corresponding evaluations were conducted. Additionally, they were provided with a tablet as a resource to complete each of the questionnaires. Prior to the assessments, they were instructed on how to complete the tests. A key limitation of the study was the reliance on self-reported measures, particularly for Tanner staging and PA questionnaires, which may introduce bias. To address this, participants were supervised by two experts in adolescent development and exercise science who provided clarification and ensured consistent interpretation of the assessment criteria. All questionnaires were completed independently under investigator supervision, with researchers available to resolve any doubts. For younger participants, a parent or guardian was present to offer information only when needed, such as recalling the frequency of specific foods at home.

The procedures of this study have been approved by the Ethical Committee for Clinical Research of the Sports Administration of Catalonia (30/CEICGC/2020, approval date: 15 December 2020) and comply with the principles and recommendations of the latest revision of the Declaration of Helsinki [19].

### 2.2. Participants

The research involved boys and girls aged 8 to 11 years (from primary school) and 12 to 16 years (from secondary school) (*n* = 668). Participants were selected from extracurricular sports programs located in the northeastern region of Spain, specifically in southern Catalonia and northern Valencia, encompassing both urban and rural settings.

The inclusion criteria were the understanding of the local language to be able to answer the questionnaire by themselves. Participants were excluded if they had any condition that contraindicated moderate-to-vigorous physical activity, including: (i) uncontrolled asthma or other chronic respiratory disease; (ii) congenital or acquired cardiovascular disorders (e.g., arrhythmia, cardiomyopathy); (iii) insulin-dependent diabetes mellitus or other metabolic disease not under medical control; (iv) musculoskeletal injury or chronic orthopaedic disorder limiting exercise; (v) neurological conditions affecting motor function; or (vi) any additional medical restriction on physical activity.

All participants and their legal guardians provided informed consent before any data were collected, in accordance with the study protocol.

A sensitivity analysis was conducted using the G*Power3 (v. 3.1.9.6) software for Mac (REFERENCE) to determine the minimum detectable effect size (*f*^2^) for the multiple regression model. With a total sample size of 668, 6 predictor variables, a significance level of α = 0.05, and a statistical power of 80% (1 − β = 0.80), the analysis indicated that the model could reliably detect effects as small as *f*^2^ = 0.02. This value corresponds to a small effect size according to Cohen’s conventional benchmarks (*f*^2^ = 0.02, small; 0.15, medium; 0.35, large), suggesting adequate sensitivity to identify modest associations in the population under study.

### 2.3. Outcomes

#### 2.3.1. Mediterranean Diet Adherence

The KIDMED questionnaire is a validated tool designed to assess adherence to the MD. It consists of 16 questions evaluating healthy and unhealthy dietary habits, with scores ranging from 0 to 12. Positive behaviors (e.g., fruit, vegetable, and fish consumption) receive +1 point, while unhealthy habits (e.g., frequent fast food or sweets) receive −1 point. Based on the total score, adherence is classified as high (≥8), moderate (4–7), or low (≤3) [14]. The KIDMED questionnaire helps identify children and adolescents at risk of poor nutrition, guiding interventions to promote healthier eating habits [9,10]. The KIDMED questionnaire, developed specifically for pediatric populations, has shown strong validity and reliability in its Spanish language version [14].

#### 2.3.2. Pittsburgh Sleep Quality Index

Sleep quality was evaluated using the Spanish version of the Pittsburgh Sleep Quality Index (PSQI) [20]. The PSQI measures different aspects of sleep over the past month, including sleep duration, disturbances, latency, and overall efficiency. It generates a global score ranging from 0 to 21, with higher scores indicating poorer sleep quality. A score above 5 is generally considered indicative of sleep difficulties.

#### 2.3.3. Physical Activity Levels

PA was evaluated using the validated Spanish versions of the Physical Activity Questionnaire for Children (PAQ-C) [21] and for Adolescents (PAQ-A) [22]. These questionnaires assess activity levels across various contexts, including physical education classes, recess, lunch breaks, after school, evenings, and weekends, as well as general activity habits. Each item is rated on a 5-point scale, with higher scores indicating greater frequency or intensity of PA. Additionally, simple questions were included to estimate the time spent on sedentary activities such as watching television, using a computer, playing video games, or sleeping. The PAQ questionnaires have shown acceptable levels of reliability and validity in youth populations when administered under suitable conditions [21,22,23,24,25].

#### 2.3.4. Maturity Status

Maturity status was assessed using a self-report questionnaire that includes a brief description and illustrative drawings of the Tanner stages [26]. This method allowed participants to identify their own stage of pubertal development by comparing their physical characteristics to standardized visual and textual descriptions. The Tanner scale, which ranges from stage I (prepubertal) to stage V (fully mature), evaluated the progression of secondary sexual characteristics such as breast and genital development in girls and boys, respectively, as well as pubic hair growth. Self-assessment of Tanner stages has been widely used in research settings as a non-invasive and practical tool for estimating pubertal status [27].

#### 2.3.5. Body Mass Index

Anthropometric measurements were taken in accordance with the standards set by the International Society for the Advancement of Kinanthropometry (ISAK) 15. Body mass was measured using a digital scale (Seca 877, Hamburg, Germany; accuracy ±0.1 kg), while height was assessed with a portable stadiometer (Seca 213, Hamburg, Germany; accuracy ±0.1 cm), and BMI was subsequently calculated. BMI was treated as a continuous variable; no categorical cut-offs were applied in order to avoid potential misclassification arising from the wide inter-individual variability in lean and fat mass among physically active adolescents. Skinfold thickness was measured using a calibrated skinfold caliper (Harpenden, Baty International, Burgess Hill, UK; accuracy ±0.2 mm) at standard anatomical landmarks. A non-elastic anthropometric tape (Lufkin W606PM, Apex Tool Group, Sparks, MD, USA) was employed for the necessary girths. Fat and muscle mass percentages were estimated using ISAK-approved equations suitable for the study population.

### 2.4. Statistical Analysis

Given the large sample size (N = 668), the Kolmogorov–Smirnov test was used to assess normality; additionally, visual inspections of Q-Q plots and residual histograms were conducted to support the evaluation. Cases with missing values were excluded from the analyses (listwise deletion). Group comparisons were conducted using Student’s *t*-test for normally distributed variables and the Mann–Whitney U test for non-normally distributed variables. Categorical variables were analyzed using the chi-square test, and post hoc analyses using adjusted residuals assessed sex differences in MD adherence.

A linear regression model was used to estimate the adherence to the MD score and its 95% confidence interval (CI), stratified by sex and adjusted for age, BMI, pubertal status, sleep quality, and physical PA levels. The KIDMED score was included as the dependent variable. Since the only categorical predictor (sex) had two levels, no multiple comparison adjustments were applied.

Model fit was evaluated using the log-likelihood ratio test, with statistical significance defined as α < 0.05. Depending on the type of variable, data are presented as mean (SD), median (Mdn) with interquartile range (IQR: Q1–Q3), or frequency.

All analyses were performed using JAMOVI for Mac [28] (version 2.6.44) and the GAMLj (version 3.5.0) module for generalized linear modeling [29].

## 3. Results

Table 1 presents the descriptive characteristics of the 668 participants studied (52 % boys). Age from 8 to 16 years, BMI from 13.10 to 34.80, waist circumference from 20.00 to 108.50 cm, fat mass from 11 to 35.60 %, and muscle mass from 17.40 to 56.90 kg. The cohort showed a mean KIDMED score of 6.2 ± 2.5 (moderate adherence), an average PAQ-C/PAQ-A index of 3.0 ± 0.6, and a median PSQI of 3 (IQR 2), indicating generally good sleep quality. Boys were slightly taller, showed more muscle mass, and had higher physical-activity indices, whereas girls had a higher percentage of body fat.

Statistically significant sex differences were reported for height (*p* = 0.043), waist (*p* < 0.001), fat mass percentage (*p* < 0.001), muscle mass (*p* < 0.001), pubertal status (*p* = 0.029), and physical PA level (*p* < 0.001).

However, post-hoc tests using adjusted residuals for pubertal status showed no significant sex differences, as the adjusted residuals for all categories (I, II, III, IV, and V) remained below the significance threshold (|z| < 1.96). These results suggest that men and women were at comparable pubertal status.

No significant sex-related differences emerged for body weight, BMI, adherence to the Mediterranean diet, or sleep quality; all comparisons yielded *p* > 0.05, indicating similar profiles for these variables in boys and girls.

The parameter estimates (coefficients) of the general linear model are shown in Table 2.

A small, borderline significant effect of the BMI variable on the participants’ levels of adherence to the MD was observed. Higher BMI levels were associated with lower levels of adherence to the MD (b = −0.21 95% CI [−0.41, −0.00], β = −0.08, t = −1.96, *p* = 0.050).

A statistically significant small effect of the Tanner variable on the participants’ levels of adherence to the MD was observed. Higher levels of maturation were associated with higher levels of adherence to the MD (b = 0.28, 95% CI [0.05, 0.51], β = 0.11, t = 2.43, *p* = 0.016).

A statistically significant small to moderate effect of the IPAQ variable on the participants’ levels of adherence to the MD was observed. Higher levels of self-reported PA were moderately linked to higher adherence to the MD (b = 0.54, 95% CI [0.33, 0.74], β = 0.22, t = 5.22, *p* < 0.001). This was the strongest predictor of MD adherence among the variables tested.

No statistically significant effects were observed for age, sex, or sleep quality on Mediterranean-diet adherence; all corresponding *p*-values exceeded 0.05.

## 4. Discussion

The present study aimed to evaluate adherence to the MD in relation to sex, age, BMI, pubertal status, sleep quality, and PA. The findings indicate significant associations between adherence to the MD and BMI, pubertal status, and PA, while no significant differences were observed concerning sex, age, or sleep quality.

### 4.1. Sex Differences and Mediterranean Diet Adherence

No statistically significant differences in MD adherence were observed between boys and girls. Despite some anthropometric and physiological differences such as height, waist circumference, fat mass percentage, muscle mass, and PA levels—sex did not play a determining role in dietary adherence. This suggests that both boys and girls have similar adherence to the MD, potentially reflecting common dietary habits influenced by environmental and cultural factors [17,31]. However, the observed anthropometric differences may be partly explained by biological factors. In particular, developmental differences, such as higher testosterone levels in males during adolescence, could contribute to increased muscle mass and lower fat mass compared to females [32], despite similar dietary patterns. Additionally, variations in PA levels can significantly impact body composition; boys who engage in more vigorous activities or sports tend to display greater muscle mass and lower fat percentages than girls, even when dietary patterns are similar [33]. Our research did not find significant differences, as previously noted in prior studies [16]. further limiting the ability to establish a clear predominance between genders.

### 4.2. BMI and Mediterranean Diet Adherence

BMI exhibited a negative association with adherence to MD, indicating that individuals with higher BMI values were less likely to adhere to the MD, potentially due to factors such as dietary preferences or barriers to accessing healthier food options. Conversely, lower adherence to the MD could contribute to weight gain, resulting in a higher BMI [13]. This finding is consistent with previous studies suggesting that unhealthy eating habits contribute to weight gain and obesity and reinforces the importance of promoting the MD as a strategy for maintaining healthy anthropometric parameters [13,31,34]. Because the BMI result sits exactly at the significance threshold, it should be viewed as tentative and confirmed in future longitudinal work.

### 4.3. Pubertal Status and Mediterranean Diet Adherence

Age did not significantly affect adherence to the MD. However, pubertal status, as measured by the Tanner stage, was significantly associated with adherence to the MD. This correlation may be attributed to several factors, such as family eating patterns or cultural environment. In Mediterranean settings, the frequency of family meals remains high throughout adolescence, and higher parental educational attainment is positively linked to MD adherence [35]. At the same time, nutrition-education programs have been shown to raise KIDMED scores [36], while participation in organized sport, which increases as puberty advances, is also associated with better adherence [34].

Our data show that more advanced pubertal stages correlated with higher adherence; this differs from previous research, where more advanced pubertal stages were correlated with lower adherence to the MD [5,16]. The contrasting studies that found lower adherence to the MD among adolescents in advanced pubertal stages could point to different methodological approaches, sample characteristics, or regional dietary norms. Specifically, the differences in sample characteristics arise because these studies involve a specific age group, focusing on adolescents at varying stages of growth and development [5]. Furthermore, the geographical variance is notable, as some studies may draw from diverse populations, leading to differences in dietary habits influenced by local practices and cultural norms surrounding the MD [16]. Nonetheless, post-hoc tests based on adjusted residuals showed no significant sex differences in pubertal stages, reinforcing that puberty itself—rather than sex—may be the key determinant in dietary behavior. Although some studies do assess maturational stage [37], to the best of our knowledge, its associations with variables such as BMI, PA, and dietary adherence had not been examined in depth. By addressing these interrelated factors within a single framework, the present study offers a more comprehensive perspective that may contribute to a better understanding of adolescent health behaviors.

### 4.4. Physical Activity and Dietary Adherence

A strong positive relationship was observed between PA levels (measured by IPAQ) and adherence to the MD. This aligns with the broader literature indicating that individuals who engage in higher levels of PA tend to adopt healthier dietary patterns [38,39]. This association may be attributed to a more health-conscious lifestyle, in which individuals prioritize both diet and exercise for overall well-being [34]. Therefore, it is important to promote PA, as we observe that it is linked to better dietary habits, particularly adherence to the MD.

### 4.5. Sleep Quality and Dietary Adherence

This dietary pattern, rich in sleep-promoting nutrients, appears to contribute to improved sleep by supporting metabolic health, reducing inflammation, and maintaining a balanced circadian rhythm [40,41]. However, no significant association was found between sleep quality (measured by PSQI) and adherence to the MD. While some studies have suggested that poor sleep quality may be linked to unhealthy eating behaviors, our findings did not support this relationship [37,42,43]. It is crucial to clarify that our study did not dismiss this assertion outright; instead, it reflects that our sample did not exhibit low levels of sleep, making it impossible to establish a definitive relationship in this context. This discrepancy may be due to sample characteristics, having an active lifestyle, or the relatively low PSQI scores in the study population, suggesting generally good sleep quality among participants. Being active, as previous research has demonstrated, is associated with improved sleep quality and reduced sleep latency, particularly during middle childhood. This connection further highlights the importance of promoting PA, as it not only contributes to better dietary habits but also positively impacts sleep patterns [44].

### 4.6. Strengths and Limitations

A major strength of this study is its large sample size (N = 668), which enhances the reliability of the findings. Additionally, the use of robust statistical analyses, including linear regression models and post-hoc residual analyses, ensures accurate interpretation of the results.

Limitations include the cross-sectional design, which prevents causal inferences, and the reliance on self-reported measures, which may introduce bias. Moreover, the convenience sample from a single Mediterranean region and the omission of family-environment variables (e.g., parental diet or socioeconomic status) may restrict generalizability.

### 4.7. Practical Implications

Higher activity and later pubertal stage coincided with better Mediterranean diet adherence. Interventions should therefore prioritize regular moderate-to-vigorous physical activity combined with concise nutrition education, focusing particularly on early pubertal adolescents and youths with elevated BMI. Embedding brief family components such as parent workshops or app-based meal-planning tools can reinforce program messages and align home meals with Mediterranean-diet principles.

## 5. Conclusions

This study identified strong correlations between adherence to the MD and BMI, pubertal status, and PA, while sex, age, and sleep quality showed no significant influence. Lower BMI was linked to greater adherence, reinforcing the diet’s role in weight management. Higher adherence in later pubertal stages suggests increased nutritional awareness, and the strong correlation with PA highlights the connection between an active lifestyle and healthy eating.

Overall, these findings highlight the intricate relationships among dietary habits, physical activity, body composition, and pubertal development in adolescents. In light of these findings, it is essential to propose targeted interventions aimed at promoting an active lifestyle. Encouraging PA among adolescents could help foster healthier eating habits, particularly adherence to the Mediterranean diet, which has been shown to contribute to better weight management and overall well-being. In addition, integrating brief, age-appropriate nutrition education modules, particularly during early puberty, when dietary awareness is emerging alongside physical-activity promotion, could further strengthen Mediterranean-diet adherence and amplify health benefits. Future research should continue to explore the multifactorial interplay between diet, PA, and other physiological factors to develop comprehensive strategies that support healthier habits among young populations.

## Figures and Tables

**Table 1:** Descriptive characteristics of the study population (Aixa-Requena et al. 2025 [30]).

Variable	All (*n* = 668)	Girls (*n* = 272)	Boys (*n* = 396)	*p* Values
**Age (years) ^a^**	11.64 ± 1.64	11.61 ± 1.66	11.66 ± 1.63	0.685
**Height (m) ^a^**	1.53 ± 0.12	1.52 ± 0.11	1.54 ± 0.13	**0.043**
**Weight (kg) ^a^**	46.74 ± 12.12	46.46 ± 11.48	46.93 ± 12.55	0.626
**BMI (kg·m^−2^) ^a^**	19.72 ± 3.19	19.91 ± 3.11	19.59 ± 3.23	0.195
**Waist (cm) ^b^**	65.50 ± 10.40	64.00± 9.50	66.10 ± 11.10	**<0.001**
**Fat mass (%) ^a^**	23.15 ± 6.12	25.52 ± 4.94	21.24 ± 6.32	**<0.001**
**Muscle Mass (kg) ^a^**	33.94 ± 8.04	32.46 ± 6.96	35.13 ± 8.65	**<0.001**
**Tanner stage, I–V ^c^**	112/209/182/128/22	37/73/83/61/11	75/136/99/67/11	**0.029**
**IPAQ (MET-min·week^−1^) ^a^**	2.99 ± 0.59	2.85 ± 0.55	3.09 ± 0.60	**<0.001**
**KIDMED (AU) ^a^**	6.24 ± 2.46	6.29 ± 2.58	6.21 ± 2.37	0.690
**PSQI, 0–21 (AU) ^b^**	3.00 ± 2.00	3.00 ± 2.00	3.00 ± 2.00	0.295

Descriptive characteristics and questionnaire results. Values in bold indicate statistically significant results (*p* < 0.05). Abbreviations: BMI: Body mass index, Waist: Waist circumference, IPAQ: International Physical Activity Questionnaire, KIDMED: Adherence to the Mediterranean Questionnaire, PSQI: Pittsburgh Sleep Quality Index. ^a^. Data are presented as mean (SD), and differences between boys and girls were examined by analysis of the variance. ^b^. Data are presented as median (IQR), and differences between boys and girls were examined by independent-samples Mann–Whitney U test. ^c^. Data are presented as frequency (%), and differences between boys and girls were examined by independent-samples chi-square test.

**Table 2:** Adherence to Mediterranean Diet (MD) in Relation to Physiological and Lifestyle Factors.

Effect	Estimate	SE	Lower 95% CI	Upper 95% CI	β	t	*p*
**(Intercept)**	6.34	0.10	6.14	6.54	0.02	62.17	<0.001
**Age (years)**	0.02	0.11	−0.20	0.25	0.01	0.21	0.836
**Sex (B–G)**	−0.37	0.21	−0.78	0.05	−0.15	−1.75	0.081
**BMI (kg·m^−2^)**	−0.21	0.11	−0.41	0.00	−0.08	−1.96	**0.050**
**Tanner stage, I–V**	0.28	0.12	0.05	0.51	0.11	2.43	**0.016**
**IPAQ (MET-min·week^−1^)**	0.54	0.10	0.33	0.74	0.22	5.22	**<0.001**
**PSQI, 0–21 (AU)**	−0.02	0.10	−0.22	0.17	−0.01	−0.23	0.816

Values in bold indicate statistically significant results (*p* < 0.05). Abbreviations: B: Boys, G: Girls, BMI: Body mass index, IPAQ: International Physical Activity Questionnaire, PSQI: Pittsburgh Sleep Quality Index.

## Data Availability

The data presented in this study are available on request from the corresponding author due to ethical restrictions related to the protection of sensitive information and privacy of underage participants, in accordance with institutional and data protection regulations.
